# *Streptococcus agalactiae* maternal colonization, antibiotic resistance and serotype profiles in Africa: a meta-analysis

**DOI:** 10.1186/s12941-019-0313-1

**Published:** 2019-03-28

**Authors:** Mucheye Gizachew, Moges Tiruneh, Feleke Moges, Belay Tessema

**Affiliations:** Department of Medical Microbiology, School of Biomedical and Laboratory Sciences, College of Medicine and Health Sciences, University of Gondar, Gondar, Ethiopia

**Keywords:** Antibiotic resistance, Colonization, GBS, Pregnant women, Recto-vaginal, Serotypes

## Abstract

**Background:**

Maternal rectovaginal colonization with *Streptococcus agalactiae* (Group B *Streptococcus* or GBS) is the most common route for the GBS disease in the perinatal period. The knowledge of maternal colonization, antibiotic resistance and serotype profiles is substantially needed to formulate the broad vaccine. However, it has not been estimated in Africa. This meta-analysis was aimed to determine the pooled prevalence of colonization, antibiotic resistance and serotype profiles of GBS reported in Africa.

**Methods:**

Potentially relevant studies from 1989 to 31th January, 2019 were retrieved from the Medline/PubMed, EMBASE, HINARI online databases, periodicals and by requesting authors. Unpublished studies retrieved from grey literature through Google and Google Scholar. Pooled estimates were calculated using the random effect model. Subgroup analysis was done to investigate the burden of colonization across sub-regions, sampling site and countries. Summary estimates were presented using words, Forest plots and Tables. Heterogeneity was assessed using the I^2^ statistic.

**Results:**

Eighty-three articles were assessed, of which 57 studies conducted in five sub-regions with 21 countries (22,206 pregnant women) met pre-specified inclusion criteria. The overall estimate of recto-vaginal colonization was 19.3% (95% CI 16.9, 21.7). The highest estimate was observed in Southern Africa, 23.8% (95% CI 18.7, 28.9), followed by Northern Africa, 22.7% (95% CI 18.2, 27.2) while the lowest was driven from the Eastern Africa, 15.4% (95% CI 12.1, 18.7). Considerable heterogeneity across and within regions, sampling site, screening methods and countries (I^2^ > 75%); and the publication bias were observed (*p *= 0.031). GBS showed the highest resistance to tetracycline. Resistance to penicillin, amoxicillin, chloramphenicol, ampicillin, ceftriaxone, ciprofloxacin, erythromycin, vancomycin and clindamycin also observed. The V, III, Ia, Ib, and II serotypes altogether were accounted 91.8% in the African studies.

**Conclusions:**

The pooled estimate of the maternal colonization with GBS was 19.3% which is equivalent with other many primary and review reports worldwide. The most antibiotic resistance estimate was recorded in the tetracycline followed by penicillin. Five serotypes were the most prevalent in Africa and more data on the antibiotic résistance and serotype distribution patterns are needed from developing countries to devise the effective preventive measures. In addition, the antibiotic susceptibility test methods used in the Africa shall be assessed for its quality.

*Trial registration* Prospero Registration Number CRD42018094525

## Background

*Streptococcus agalactiae* (or *S. agalactiae* or Group B *Streptococcus;* GBS) is one of the many serologically distinct species within the genus *Streptococcus* [1, 2]. It is an encapsulated diplococcus exhibiting ß-haemolysis on blood agar, facultative anaerobe, nutritionally fastidious, catalase, and mannitol salt negative. It also hydrolyzes sodium hippurate, bacitracin resistant, CAMP test positive and chain forming group. It is found as a commensal organism in the gut and genital tract of both female and male healthy adults. It causes severe illnesses in people of all ages, ranging from bloodstream infections (sepsis) and pneumonia to meningitis and skin infections [1, 3]. It also causes a significant agricultural and veterinary problem, since it can infect the ruminants` mammary glands [4], and fishes [5].

In the 1970s, GBS was the dominant pathogen in the early neonatal period [6]. It also became the most common cause of neonatal sepsis and meningitis in many developed countries in the early 1980s [7]. Newborns from GBS colonized mothers could be exposed in utero, or during delivery as they swallow or aspirate the bacterium while passing through the birth canal. GBS infection in infants causes sepsis and meningitis which could result in acute illness, long-term disabilities and death [8]. Isolates from human express capsular polysaccharide (CPS), a major virulence factor that helps the bacterium to evade the host defense mechanisms [9].

Primary studies conducted in the East African countries showed the colonization rates ranged from 3.0% to 28.8% [10–26]; Central Africa, 20.0% [27, 28]; Western Africa, 2.5% to 34.2% [29–48]; Southern Africa, 1.77% to 48.23% [49–61]; and Northern Africa, 17.00% to 26.5% [62–64]. GBS isolated from pregnant women in different primary studies conducted in Africa showed resistance to penicillin, ampicillin, erythromycin, clindamycin, vancomycin, ciprofloxacin, chloramphenicol, and tetracycline [10, 11, 15, 31, 36, 65]. Unlike to Western countries, few data are available about GBS serotypes in different parts of the Africa since the 1989 in Ethiopia to 2018 in Morocco [22, 28, 30, 31, 33, 39, 49, 54, 66]. A review from the USA on GBS serotypes showed lower proportions of women with serotypes Ia, Ib, or III with the mean prevalence estimate of 55.0%, and in Europe, 58.3% [67].

A systematic review done 10 years ago on 21 studies included 24,093 women from the 13 European countries indicated that GBS colonization varied from 6.5% in Turkey to 36% in Denmark [68]. Another recent review, based on the studies using the recommended methods, estimated the maternal GBS prevalence as 17.9% worldwide, ranging from 11.1% in Southeast Asia to 22.4% in Africa [67]. Such a review included 78 primary studies from the 37 countries with main limitations in Africa and Asia. Another meta-analysis study included 390 articles from 85 countries with a total of 299, 924 pregnant women showed 18% overall global estimates of maternal GBS colonization, with regional variation from 11.1 to 34.7%, and lower prevalence in Southern Asia, 12.5% and Eastern Asia, 11% [69].

Reviews of the prevalence estimate of pregnant women colonized with GBS, antibiotic resistance profile and serotype distribution are useful to generate evidence and to devise the preventive measures. Thus, this review was aimed to estimate the pooled prevalence of maternal colonization with GBS, antibiotic resistance and serotype patterns reported from various studies conducted in African countries.

## Methods

### Identification and selection of studies

Published and unpublished research reports describing GBS maternal colonization, antibiotic resistance profile and serotype distribution in Africa since 1989 to 31th January, 2019 were reviewed. Potentially relevant studies were identified through a literature search of PubMed/Medline, HINARI, and EMBASE online databases; from periodicals to requesting articles from publishers/authors. Unpublished studies were retrieved from the grey literature through Google and Google Scholar. All searches were limited to English language and conducted from February 2018 to January 2019. The phrase ‘*Streptococcus agalactiae’* was searched following a combination of free text and thesaurus terms in different variations: Group B Streptococcus, GBS, Streptococci, maternal, pregnancy, parturient, third trimester, colonization, carriage, vaginal, rectal, vaginorectal, rectovaginal, prevalence, proportion, antibiotic/drug/antimicrobial, resistance/susceptibility patterns/profiles, serotype, serotype distribution, and Africa. The following keywords were used to retrieve studies from PubMed database; (*Streptococcus agalactiae*) AND (maternal AND colonization OR (parturient AND prevalence AND proportion)) AND (antibiotic/antimicrobial susceptibility/resistance AND serotype OR (drug AND resistance)) AND (Africa). The search was carried out by three authors (MG, MT, & FM), the most relevant studies were selected using predefined inclusion and exclusion criteria. The last author (BT) has checked the overall consistency of the searching process, study choice and inclusion/exclusion criteria.

**Fig. 1:**
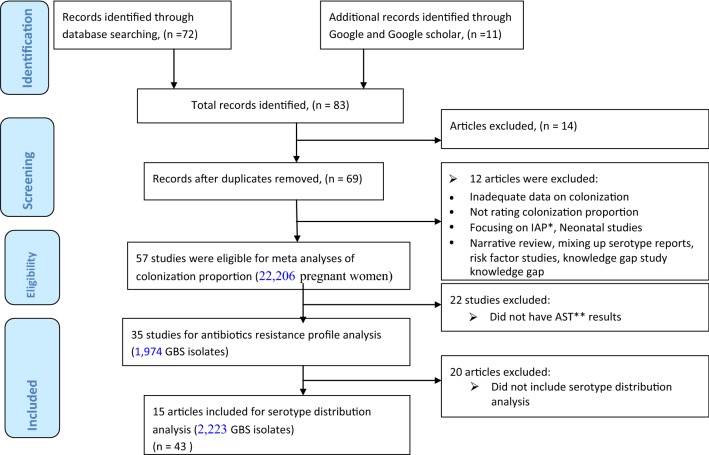
Flow chart indicating the result of literature search (*Intrapartum antibiotic prophylaxis, **antibiotic susceptibility test)

Abstracts were reviewed from a first search using predefined inclusion and exclusion criteria. Original studies from the African settings were included in this systematic review and meta analysis study, whereas comments, editorials, and reviews were excluded. The articles were included if they estimated the proportion/prevalence/carriage and/or antibiotic resistance patterns and/or the serotype profiles among the pregnant women colonized with GBS; excluding those colonized mothers for whom proportion of colonization were not reported. The review was carried out by using the Preferred Reporting Items for Systematic reviews and Meta-Analyses (PRISMA) guideline (Fig. 1) [70] records after duplicates were removed.

### Data extraction

Two authors (MG, FM) performed data abstraction using excel spreadsheet form. These authors independently examined titles, abstracts, full-text articles, and abstracted data using the same data abstraction forms and selection criteria from studies conducted on maternal GBS colonization in Africa since 1989 to 31th January, 2019. Disagreements were resolved by consensus among these investigators. The third and fourth authors, MT and BT arbitrated any discrepancies between the two authors who primarily abstracted the data. From each study, the following parameters were extracted: numbers of pregnant women involved in the study, culture methods used, specimen collection site, colonization (GBS positive), antibiotic resistance and serotype profiles of the isolates. Moreover, the authors retrieved data on study country, sub-region/continent, study year, and study design. Ethical approval for this review was not applicable.

### Validity assessment

Studies were assessed for quality, with moderate to high quality studies included in the analysis. The quality of included studies was assessed by using the Newcastle–Ottawa quality assessment scale [71]. Two authors (MG, MT) independently assessed the methodological quality, quality of reported data (extractable data to calculate colonization proportion, antibiotic resistance profile and serotype distribution and cleared data research design of the included studies. After assessing the quality of each study included on the basis of these criteria, a composite quality score was assigned, ranging from 0 to 7. Studies scoring 5 and above were judged to be of moderate to high quality.

### Data analysis

The data extracted were entered into the Microsoft excel spreadsheet and were exported to the STATA version 14 (Stata Corp LLC, Texas, USA) for analysis. The magnitude of heterogeneity between the included studies was quantitatively measured by an index of heterogeneity (I^2^ statistics) [72]. The low, medium and high heterogeneity were represented as the I^2^ values of 25%, 50% and 75%, respectively. The statistical significance of heterogeneity was determined by a p-value of I^2^ statistics. A *p*-*value* ≤ 0.05 statistically showed heterogeneity. If I^2^ value was greater than 50%, we used Dersimonian and Liard random effect model to determine the pooled estimates of GBS colonization proportion, antibiotics resistance and serotype profiles of the isolate [73]. The subgroup analysis was conducted by considering sub-regions, countries, specimen collection sites and method of GBS screening used as a grouping variable (Table 1). Small-study effects and publication bias were evaluated first visually by using the funnel plot (Fig. 4), and then by Egger’s statistics in the random effect model (Table 2). The *p*-*value *≤ *0.05* was considered indicative of the presence of statistically significant publication bias [74–77] quantified. The trim and fill method was used to correct the publication bias as indicated in Fig. 5. The results were presented in text, tables, funnel and Forest plots.

**Table 1:** Meta-analysis of studies reporting proportion of maternal colonization with GBS in African by Sub-regions, specimen collection site, method of GBS screening used and countries since 1989 to 31th January 2019

Sub-regions	No. of countries	No. of studies	No. of women	No. of GBS isolates	Estimated proportion (95% CI)	I^2^(%)^a^	p-value
Eastern Africa	5	17	5874	927	15.397 (12.119, 18.674)	92.3	0.000
Central Africa	2	2	1058	211	19.943 (17.540, 22.346)	0.0	0.000
Western Africa	6	20	5426	1183	18.704 (13.914, 23.494)	95.9	0.000
Southern Africa	5	14	8849	2019	23.773 (18.681, 28.865)	97.2	0.000
Northern Africa	3	4	1099	224	22.671 (18.162, 27.179)	65.5	0.000
Total	21	57	22,206	4564	19.328 (16.972, 21.684)	95.6	0.000
Specimen collection site							
Rectovaginal	–	41	16,136	3393	20.345 (17.459, 23.231)	95.9	0.000
Vaginal	–	16	6070	1171	16.674 (12.415, 20.932)	94.8	0.000
Total		57	22,206	4564	19.328 (16.972, 21.684)	95.6	0.000
Screening methods							
Direct plating	–	20	6114	924	14.698 (11.411, 17.984)	94.5	0.000
Prior broth enrichment	–	35	15,733	3544	21.827 (18.868 24.786)	95.0%	0.000
Rapid test	–	2	395	97	23.191 (10.741, 35.642)	79.5%	0.000
Total	–	57	22,206	4564	19.328 (16.972, 21.684)	95.6	0.000
Countries							
Ethiopia	–	11	4428	667	14.702 (11.783, 17.621)	85.0	0.000
Sudan	–	1	50	8	16.000 (5.838, 26.162)	–	0.002
Kenya	–	2	492	66	11.656 (− 5.539, 28.852)	97.7	0.184
Tanzania	–	2	595	97	16.135 (2.899, 29.372)	95.2	0.017
Uganda	–	1	309	89	28.803 (23.746, 33.859)	–	0.000
Gabon	–	1	549	109	19.854 (16.522, 23.186)	–	0.000
DRC	–	1	509	102	20.039 (16.570, 23.508)	–	0.000
Ghana	–	3	1019	223	21.660 (15.857, 27.462)	77.1	0.000
Gambia	–	3	1247	397	29.810 (23.920, 35.701)	77.1	0.000
Nigeria	–	10	2078	433	18.238 (12.363, 24.112)	92.4	0.000
Togo	–	1	200	5	2.500 (0.344, 4.656)	–	0.023
Cameron	–	2	242	30	12.276 (8.145,16.407)	56.3	0.001
Benin	–	1	640	100	15.625 (12.803, 18.447)	–	0.000
South Africa	–	6	4158	1067	30.389 (22.250, 38.527)	96.1	0.000
Mozambique	–	2	433	70	11.415 (− 7.674, 30.504)	98.2	0.241
Zimbabwe	–	3	1444	359	25.851 (17.581, 34.121)	92.4	0.000
Namibia	–	1	860	117	13.605 (11.313, 15.896)	–	0.000
Egypt	–	2	350	91	25.992 (21.398, 30.585)	0.0	0.000
Morocco	–	1	349	82	23.496 (19.047, 27.945)	–	0.000
Tunisia	–	1	300	51	17.000 (12.747, 21.253)	–	0.000
Malawi	–	2	1954	406	20.225 (16.889, 23.561)	25.6	0.000
Total	–	57	22,206	4564	19.328 (16.972, 21.684)	95.6	0.000

*DRC* Democratic Republic of Congo, *CI* confidence interval

^a^Chi-square

**Table 2:** Proportion of GBS isolated from African pregnant women by Sub-regions, screening methods and specimen types used

Sub-region (#5)	Number of studies, methods used and number of isolates							Specimen and number of isolates			
	No. studies	Method						Specimen			
		Prior broth, N (%)	Isolates, N (%)	Direct plate, N (%)	Isolates, N (%)	Rapid, N (%)	Isolates, N (%)	Vaginal, N (%)	Isolates. N (%)	RV, N (%)	Isolates, N (%)
East Africa	17	8 (47.1)	448 (48.3)	7 (41.2)	382 (41.2)	2 (11.8)	97 (10.5)	4 (23.5)	90 (9.7)	13 (76.5)	837 (90.3)
West Africa	20	14 (70.0)	921 (77.9)	6 (30.0)	262 (22.1)	0 (0.0)	0 (0.0)	8 (40.0)	304 (25.7)	12 (60.0)	879 (74.3)
Central Africa	2	0 (0.0)	0 (0.0)	2 (100.0)	211 (100.0)	0 (0.0)	0 (0.0)	1 (50.0)	102 (48.3)	1 (50.0)	109 (51.7)
South Africa	14	11 (78.6)	1730 (85.7)	3 (21.4)	289 (14.3)	0 (0.0)	0 (0.0)	2 (14.3)	637 (31.6)	12 (85.7)	1382 (68.4)
North Africa	4	2 (50.0)	91 (40.6)	2 (50.0)	133 (59.4)	0 (0.0)	0 (0.0)	1 (25.0)	38 (17.0)	3 (75.0)	186 (83.0)
Total	57	35 (61.4)	3190 (70.0)	20 (35.1)	1277 (28.0)	2 (3.5)	97 (2.0)	16 (28.1)	1172 (25.7)	41 (71.9)	3392 (74.3)
Countries											
Ethiopia	11	7 (63.6)	379 (56.8)	4 (36.4)	288 (43.2)	0 (0.0)	0 (0.0)	3 (27.3)	82 (12.3)	8 (72.7)	585 (87.7)
Sudan	1	0 (0.0)	0 (0.0)	0 (0.0)	0 (0.0)	1 (100.0)	8 (100.0)	1 (100.0)	8 (100.0)	0 (0.0)	0 (0.0)
Kenya	2	0 (0.0)	0 (0.0)	2 (100.0)	66 (100.0)	0 (0.0)	0 (0.0)	0 (0.0)	0 (0.0)	2 (100.0)	66 (100.0)
Tanzania	2	1 (50.0)	69 (71.1)	1 (50.0)	28 (28.9)	0 (0.0)	0 (0.0)	0 (0.0)	0 (0.0)	2 (100.0)	97 (100.0)
Uganda	1	0 (0.0)	0 (0.0)	0 (0.0)	0 (0.0)	1 (100.0)	89 (100.0)	0 (0.0)	0 (0.0)	1 (100.0)	89 (100.0)
Gabon	1	0 (0.0)	0 (0.0)	1 (100.0)	109 (100.)	0 (0.0)	0 (0.0)	0 (0.0)	0 (0.0)	1 (100.0)	109 (100)
DRC	1	0 (0.0)	0 (0.0)	1 (100.0)	102 (100.0)	0 (0.0)	0 (0.0)	1 (100.0)	102 (100.0)	0 (0.0)	0 (0.0)
Ghana	3	3 (100.0)	223 (100.0)	0 (0.0)	0 (0.0)	0 (0.0)	0 (0.0)	0 (0.0)	0 (0.0)	3 (100.0)	223 (100.0)
Gambia	3	2 (66.7)	283 (71.3)	1 (33.3)	114 (28.7)	0 (0.0)	0 (0.0)	2 (66.7)	144 (36.3)	1 (33.3)	253 (63.7)
Nigeria	10	9 (90.0)	415 (95.8)	1 (10.0)	18 (4.2)	0 (0.0)	0 (0.0)	3 (30.0)	44 (10.2)	7 (70.0)	389 (89.8)
Togo	1	0 (0.0)	0 (0.0)	1 (100.0)	5 (100.0)	0 (0.0)	0 (0.0)	1 (100.0)	5 (100.0)	0 (0.0)	0 (0.0)
Cameroon	2	0 (0.0)	0 (0.0)	2 (100.0)	25 (100.0)	0 (0.0)	0 (0.0)	1 (50.0)	11 (44.0)	1 (50.0)	14 (56.0)
Benin	1	0 (0.0)	0 (0.0)	1 (100.0)	100 (100.0)	0 (0.0)	0 (0.0)	1 (100.0)	100 (100.0)	0 (0.0)	0 (0.0)
South Africa	6	6 (100.0)	1067 (100.0)	0 (0.0)	0 (0.0)	0 (0.0)	0 (0.0)	1 (16.7)	551 (51.6)	5 (83.3)	516 (48.4)
Mozambique	2	1 (50.0)	2 (2.9)	1 (50.0)	68 (97.1)	0 (0.0)	0 (0.0)	0 (0.0)	0 (0.0)	2 (100.0)	70 (100.0)
Zimbabwe	3	2 (66.7)	273 (76.0)	1 (33.3)	86 (24.0)	0 (0.0)	0 (0.0)	1 (33.3)	86 (24.0)	2 (66.7)	273 (76.0)
Namibia	1	1 (100.0)	117 (100.0)	0 (0.0)	0 (0.0)	0 (0.0)	0 (0.0)	0 (0.0)	0 (0.0)	1 (100.0)	117 (100.0)
Malawi	2	1 (50.0)	390 (96.1)	1 (50.0)	16 (3.9)	0 (0.0)	0 (0.0)	0 (0.0)	0 (0.0)	2 (100.0)	406 (100.)
Egypt	2	2 (100.0)	91 (100.0)	0 (0.0)	0 (0.0)	0 (0.0)	0 (0.0)	1 (50.0)	38 (41.8)	1 (50.0)	53 (58.2)
Morocco	1	0 (0.0)	0 (0.0)	1 (100.0)	82 (100.0)	0 (0.0)	0 (0.0)	0 (0.0)	0 (0.0)	1 (100.0)	82 (100.0)
Tunisia	1	0 (0.0)	0 (0.0)	1 (100.0)	51 (100.0)	0 (0.0)	0 (0.0)	0 (0.0)	0 (0.0)	1 (100.0)	51 (100.0)
Total	57	35 (61.4)	3309 (72.5)	20 (35.1)	1158 (25.4)	2 (3.5)	97 (2.1)	16 (28.1)	1171 (25.7)	41 (71.9)	3392 (74.3)

*DRC* Democratic Republic of Congo

#### Outcome of interest

The major outcome of interest of this review was the pooled proportion of GBS colonization of pregnant women, antibiotic resistance profiles and serotype patterns of the isolates reported from different studies in Africa. Sub-group analysis was done by sub-regions (Northern Africa, Western Africa, Central Africa, Eastern Africa and Southern Africa), and the 21 countries as detailed in Table 1. The proportion of resistance GBS to the 10 different antibiotics was calculated by dividing the numbers of resistance isolates by the total number of GBS isolated from pregnant women. The proportion of 10 capsular type patterns of GBS was also carried out by using the methods which we applied for the estimate analysis of antibiotic resistance proportion.

## Results

**Fig. 2:**
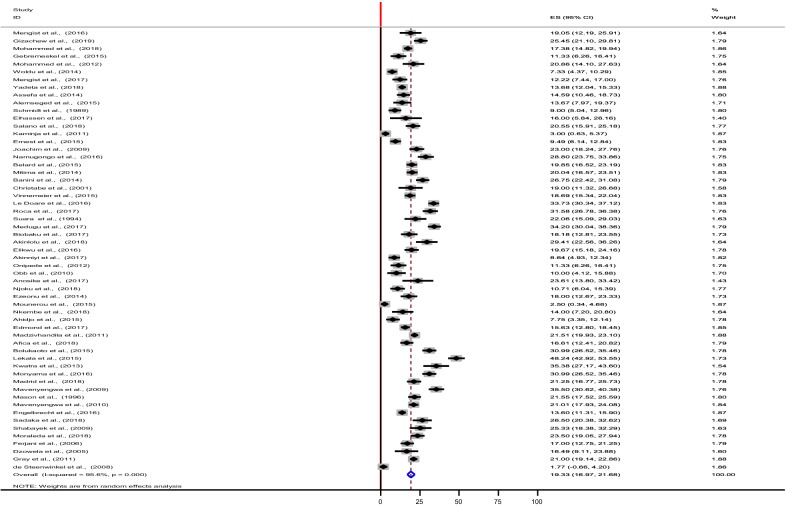
Forest plot showing the pooled estimates of the maternal GBS colonization proportion in Africa

This meta-analysis study pooled the colonization, antibiotic resistance profiles and the serotype distributions of GBS isolates which have investigated in small and fragmented ways. As shown in Fig. 1, 57 studies were identified from the five sub-regions of the African continent. These studies included 22,206 pregnant women for the estimation of maternal GBS colonization proportion, 1974 GBS isolates were tested for antibiotic susceptibility profiles, and 2223 GBS isolates were analyzed for serotype distribution. The pooled estimate of the maternal GBS colonization proportion in this study was 19.3% (95% CI (16.9, 21.7) (Table 1, and Fig. 2).

Among the 22,206 pregnant women included in 57 studies across the 21 countries, 4564 (3393 rectovaginal and 1171 vaginal) pregnant women were colonized with GBS (Tables 1, 2).

**Fig. 3:**
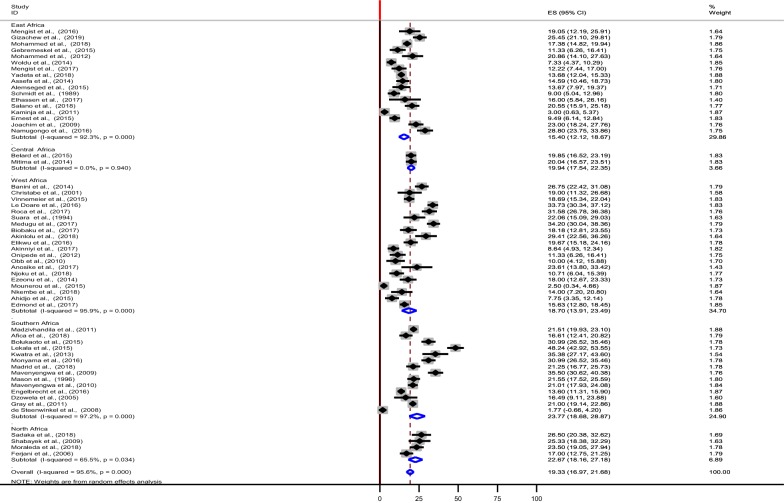
Forest plot showing the Regional pooled estimates of the maternal GBS colonization proportion in Africa

Considerable heterogeneity was observed in this meta-analysis (I-squared, 95.6%). To find the possible source (s) of variability between the included studies in this review, sub-group analysis was done by using five sub-regions, the 14 studies from the five Southern African countries had the highest number of pregnant women (n = 8849) participated in the study while the two studies conducted in the Central African countries had (n = 1058) the lowest number of the study participants. The overall mean proportion estimates of 19.3% (95% CI 16.9, 21.7) were slightly similar to the estimate derived from the Central African studies 19.9% (95% CI 17.5, 22.3) (Table 1 and Fig. 3). In addition, the highest colonization proportion was estimated from studies compiled in the Southern African countries, 23.8% (95% CI 18.7, 28.9), followed by studies conducted in Northern African courtiers, 22.7% (95% CI 18.2, 27.2). While the least estimate of maternal GBS colonization proportion was observed from the East African studies, 15.4% (95% CI 12.1, 18.7) (Table 1 and Fig. 3). Among the GBS screening techniques used in studies conducted in the African countries, the rapid test method accounted the highest estimate (23.2%, 95% CI 10.7, 35.6) though the estimate was derived from two studies while the direct plating techniques had the lowest estimation (14.7%, 95% CI 11.4, 17.9) (Table 1).

As detailed in Table 2, the percentage of laboratories employed by the primary author (s) were detailed by the five sub-regions, samples used and 21 countries where the 57 studies (primary articles) compiled to assess whether the differences or heterogeneity of colonization prevalence observed are attributable to geographical, methodological or sample types used differences. South Africa among the 14 studies, 11 (78.6%) used the broth enrichment techniques prior to inoculating on to solid media, followed by West Africa, 14 (70.0%). Studies from Central Africa used direct plating method. In all sub-regions, the primary authors used more recto-vaginal samples for GBS screening, and the highest estimate was recorded among the Sothern African countries, 12 (85.7%), followed by the East Africans, 13 (76.5%).. Six countries which contributed ≥ 3 articles had 36 (63.2%) article coverage for this study. Of these countries, South Africa and Ghana used 100% enrichment broth followed by Nigeria (90.0%). Twelve studies from 10 countries failed to use the prior enrichment techniques for GBS screening, and four studies collected from four countries also did not use recto-vaginal samples. Table 2 also showed us that the sub-regions which used the prior enrichment broth (70.0%) and recto-vaginal samples (74.3%) had better detection rates of GBS. It was also reflected in the countries at which more GBS was recovered by using prior enrichment broth (72.5%), and recto-vaginal sample (74.3%).

**Fig. 4:**
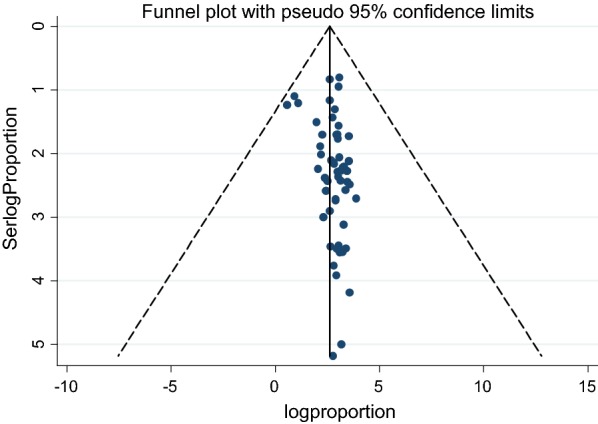
Funnel plot showing symmetrical distribution of the studies (visually no publication bias)

Further more, small study effect (or publication bias) was observed in this review as it is shown in the funnel plot (Fig. 4) and Egger’s statistical test (*p*-*value* = *0.031*) (Table 3).

**Table 3:** Tests for publication bias of the meta-analysis studies of maternal colonization with GBS in the Africa since 1989 to 31th January, 2019

Begg’s test						
Adj. Kendall’s score (P − Q)	43					
Std. dev. of score	145.26 (corrected for ties)					
Number of studies	57					
z	0.30					
Pr > |z|	0.767					
z	0.29 (continuity corrected)					
Pr > |z|	0.772 (continuity corrected)					
Egger’s test						
Number of studies	57					
Root of MSE	0.3666					

Std_eff	Coef.	Std. err.	t	P > |t|	[95% conf. interval]	
Slope	2.13663	2.378439	8.98	0.000	1.65998	2.61328
Bias	2.853829	1.287509	2.22	0.031	0.273604	5.434055

Test of H0: small-study effects exist as the *p-value *= 0.031

*MSE* Mean square error

**Fig. 5:**
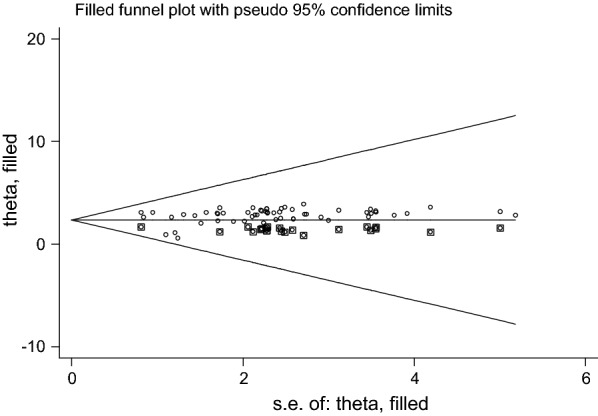
Meta-trim funnel; the “trim and fill” method to adjust for publication bias in funnel plot that shows symmetrical distribution of the studies

Thus, trim and fill method was used to correct publication bias observed in our meta-analysis and the corrected symmetric graph is indicated in Fig. 5.

### Antibiotic resistance profiles of Group B Streptococcus

As detailed in Fig. 1 and Table 4, of the 57 studies collected from 21 African countries, the 35 studies reported the antibiotic resistance patterns of GBS among the 1974 isolates obtained from pregnant women. The highest pooled proportion of antibiotic resistance was observed in Tetracycline, 82.6% (95% CI 75.9, 89.4), followed by penicillin, 33.6% (95% CI 17.0, 50.1).

**Table 4:** Pooled proportion of antibiotic resistance GBS isolated from pregnant women in Africa since 1996 to 31th January 2019

Antibiotics disc tested for GBS^a^	No. of Studies reporting antibiotic resistance (n = 34)	Estimated proportion of antibiotic resistance (95% CI)	I^2^ (%)^a^	p-value
Penicillin	9	33.562 (17.027, 50.096)	97.1	*0.000*
Ampicillin	8	26.783 (10.709, 42.857)	95.1	*0.001*
Clindamycin	22	19.632 (14.350, 24.915)	91.7	*0.000*
Erythromycin	27	20.823 (15.610, 26.036)	91.5	*0.000*
Vancomycin	9	19.702 (10.206, 29.199)	89.1	*0.000*
Ceftriaxone	12	25.993 (17.350, 34.637)	84.3	*0.000*
Amoxicillin	2	31.422 (22.783, 40.060)	0.0	0.000
Ciprofloxacin	8	24.564 (10.789, 38.339)	94.7	*0.000*
Chloramphenicol	9	27.339 (15.844, 38.835)	96.2	*0.000*
Tetracycline	13	82.626 (75.899, 89.354)	95.2	*0.000*

Italic values indicate significance of p-value (p < 0.05)

*CI* Confidence interval

^a^Chi square

### Serotype distribution

Of the 57 articles reviewed, 15 studies had serotype analysis of GBS in Africa from the dataset (Fig. 1). At least one of the ten serotypes was identified among these 15 studies included in this review, including the non-type-able (NT) isolates in nine studies. The pooled proportion of serotypes of maternal GBS analyzed from the five or more studies indicated that serotype V, III, Ia, Ib and II, accounted 91.8% coverage in the African reports (Table 5) that reported in ten or more studies. These serotypes were predominant in the African setting each with: 29.2% (95% CI 19.8, 38.6), 19.7% (95% CI 10.9, 28.5), 17.6% (95% CI 11.9, 23.4), 15.6% (95% CI 10.7, 20.5), and 12.4% (95% CI 8.4, 16.4) respectively.

**Table 5:** Pooled proportion of serotype distributions among GBS isolated from pregnant women in Africa since 1989 to 31th January 2019

Serotypes	No. of studies reporting serotypes	No. of isolates reported for each serotype	Estimated proportion of each serotype (95% CI)	I^2^ (%)^a^	p-value
Ia	15	426	17.644 (11.878, 23.409)	97.7	0.000
Ib	10	188	15.601 (10.732, 20.469)	90.3	0.000
II	15	307	12.367 (8.373, 16.361)	95.4	0.000
III	15	547	19.717 (10.949, 28.484)	98.2	0.000
IV	9	64	2.587 (1.184, 3.990)	89.0	0.000
V	14	591	29.226 (19.838, 38.615)	98.4	0.000
VI	3	33	16.013 (2.307, 29.719)	96.8	0.022
VII	3	71	30.030 (− 4.383, 64.442)	97.0	0.087
VIII	2	30	24.418 (− 11.682, 60.518)	96.2	0.185
IX	4	64	20.977 (4.953, 37.001)	96.3	0.01
NT	9	156	7.012 (3.379 10.645)	95.5	0.000
Other^b^	2	9	1.614 (− 1.027, 4.256)	81.1	0.231

*CI* confidence interval

^a^Chi square

^b^A strain with mixed serotype and a strain with a unique random fragment length polymorphism pattern (cpsV variant)

## Discussion

So far the prevalence of maternal colonization with GBS, antibiotic resistance profiles and serotype distributions of the isolates in the African setting is investigated in small and fragmented ways. Therefore, this is the first meta-analysis of its kind to summarize the pooled proportion of maternal recto-vaginal GBS colonization reported in 57 studies among 21 countries. Hence, in the present analysis, the pooled estimate of the colonization proportion was 19.3% (95% CI (16.9, 21.7) with the sub-regional variation of 14.0% (95% CI 10.41, 17.60) in Eastern Africa to 23.8% (95% CI 18.7, 28.9) in the Southern Africa (Table 1). Finding of the current meta-analysis is almost comparable with the meta-analysis study conducted worldwide in the 2016 which had 17.9% (95% CI 16.2, 19.7) overall estimates of maternal rectovaginal GBS colonization proportion from 78 studies with 73,791 pregnant women [67]. However, in the sub-group analysis of such a global estimate of the maternal rectovaginal colonization, the Africa represented in only four studies involving 2735 participating women with 619 GBS positive took the highest estimate of colonization proportion, 22.4% compared to the other sub-groups in such a review. This pooled result is slightly higher than the overall estimate of the colonization proportion of the current meta-analysis. This discrepancy might be explained by the variability in the number of the studies involved in the meta-analysis, the number of pregnant women participated in between the two reviews, variations in the detection techniques (laboratory facilities) employed and biological factors among the study participants across the world.

Another meta-analysis study analyzed the dataset about maternal colonization included 390 articles, 85 countries, and a total of 299, 924 pregnant women found the worldwide adjusted estimate for maternal GBS colonization was 18% (95% CI 17–19), with the regional variations from 11.1% (95% CI 9.9–12.4) in the Eastern Asia to 34.7% (95% CI 29.5–39.9) in the Caribbean. In the same meta-analysis study, Africa represented in the 19 studies included 36,130 pregnant women with the reported prevalence rate of 18.2% (95% CI 16.1–20.4) and overall pooled estimate of the adjusted colonization proportion was 21.3% (95% CI 18.5–24.2) [69] which is in consistent with our estimate. In the Russell et al., review, the lowest estimate was recorded from the six studies analyzed from the Western Africa, 17.5% (95% CI 10.8–24.1) while the highest estimate was from the Southern Africa, 28.9% (95% CI 26.6–31.2). The current systematic review and meta-analysis which we analyzed also reaffirmed that the highest pooled proportion of maternal colonization was derived from the 14 studies compiled in the Southern African countries (23.8%) while the lowest estimate was from the 17 studies recorded from the Eastern African countries (15.4%) where higher estimate (19.4%) was made in the previous study [69]. The mean proportion of maternal colonization with GBS estimated in our study from West Africa (18.7%) was the same as the estimate of the previous review in the same (17.5%) sub-region analysis [69]. Furthermore, findings of the current review in Africa is lower than a sub-analysis of the systematic review done 10 years back on 21 studies presented data on 24,093 women from 13 European countries which indicated that GBS colonization was varied from 6.5% in Turkey, to 36% in Denmark [68]. In such a report, colonization estimate from Denmark is higher than the estimate of our meta-analysis, whereas the estimate from Turkey is lower than the estimate of the current study.

The possible reason for the inconsistency of the estimates between sub-regions might be associated with differences in the numbers of the studies (articles) analyzed (Table 2), variations in the number of the study participants (pregnant women) included in the studies, differences in GBS detection techniques and sample types (Table 2) used across the sub-regions where certain laboratories use different alternative tests available like prior enrichment broth or rapid tests while others use routine or traditional (direct plating of the swabbed specimen onto solid media) laboratory techniques, disparities in site of specimen collection, and biological factors of the study participants. This is supported by the findings of this study to which to find the possible sources of heterogeneity, sub-group analysis was conducted by considering sub-regions, site of specimen collections (type of samples), the laboratory methods used for and the countries where studies collected as a grouping variable and the result showed that there was considerable heterogeneity (Table 1) in most cases (I^2^ > 75%; p-value ≤ 0.05). The rapid GBS screening method gave the highest pooled estimate prevalence of pregnant women colonization with GBS (23.2% (95% CI 10.7, 35.6), but the result was estimated from the two reports (one was screened by PCR and the other one was by rapid test kit). Based on Table 2 details, we realized that use of prior enrichment broth and recto-vaginal samples contributed for the variability of the colonization prevalence estimates. In addition, the number of articles compiled may have an effect on this heterogeneity. Since the possible source of variations might be numerous, further studies are required to identify specific aspects of season, co-morbidities, ethnicity, genetics/biological factors, lifestyle, behavior or cultural practices that may be factors for increase in the prevalence of GBS in different geographical locations.

The widespread IAP uses to prevent early onset GBS disease has raised a concern about the emergence of antibiotic resistance among GBS isolates. GBS continues to be susceptible to penicillin, ampicillin, and first-generation cephalosporins [78–81]. However, the isolates with increasing minimum inhibitory concentrations (MICs) to penicillin or ampicillin have been reported in both the noninvasive [82] and invasive isolates [83]. A report revealed that a penicillin-binding protein (PBP2X) alterations were found in noninvasive GBS isolates [82]. Correspondingly, the pooled finding of the current review recorded the presence of a concern about the antibiotic resistance of GBS isolated from the pregnant women. The isolates exhibited resistance to penicillin, ampicillin, vancomycin, clindamycin and erythromycin, which are usually recommended for the IAP to pregnant women at near (4 h before) delivery [3]. Similarly, in a 2017 study conducted in USA, a minimum inhibitory concentration technique was used for susceptibility profile by micro-dilution test, showed the presence of 28 GBS isolates resistant to the six beta-Lactam antibiotics [84] though the prevalence varies.. In another study conducted in 2014, two isolates sent to the CDC Streptococcus Laboratory were confirmed as vancomycin resistant and both were grouped as capsular serotype II, multilocus sequence type 22 GBS [85]. In our review, the capsular serotype II is one among the dominant serotypes in the African setting.

Occurrence of resistant GBS isolates to the bet-lactam antibiotics and vancomycin in the low income countries including Africa is possibly because of the widespread use of the antibiotics empirically for the treatment of different infectious diseases and the availability of these drugs non-restrictively in different areas with lower price enable self prescription. This expanded use of the beta-lactam antibiotics in the treatment of several infective clinical syndromes and the easy of purchase over the counter might be the contributing causes for the selective pressure for the emergence of GBS resistance strains in the area. The resistant rates to vancomycin reported in different African countries also might have an epidemiologic link with serotypes II and sequence type 22 which needs further studies by using the high tech laboratory facilities. In addition, there is a need to devise a system that could help, at least, to decrease the irrational use of these antibiotics in Africa. From this review, we realized that the presence of resistance GBS to penicillin, ampicillin and vancomycin in the African studies is not uncommon in contrary to the reports from the western countries. Thus, evaluating the quality of GBS antimicrobial testing methods and the quality of the antimicrobial disks used including shipment and storage in the developing continents shall be taken into consideration.

Knowing the serotype distribution of GBS is vital to understand the epidemiology of GBS infections. Currently, a total of 10 distinct GBS capsular serotypes (Ia, Ib, and II–IX) have characterized according to the capsular polysaccharide (CPS), one of the major known virulence factors underlying invasive GBS disease [86]. The five serotypes such as Ia, Ib, II, III, and V are the most common which accounted for more than 85% of serotypes in the global regions that have reported serotype data, including the Americas (96%), Europe (93%), and the Western Pacific (89%) [87]. Correspondingly, in our meta-analysis, the five serotypes: V, III, Ia, Ib and II were the most common which accounted 91.8% among the 10 serotypes analyzed in the 21 African countries. In addition, the estimated mean prevalence of the serotypes Ia, Ib, or III reported in studies conducted in the USA and Europe were 55.0% (95% CI 52.3, 57.7), and 58.3% (95% CI 52.2, 64.5), respectively. It is slightly in agreement with our finding in which these three serotypes (Ia, Ib and III) accounted 62.4%. We also found that IX accounted 20.9% (95% CI 4.9, 37.0) compiled from four studies, and 7.0% (95% CI 3.4, 10.6) non-typeable (NT) GBS analyzed from the nine studies which could potentially be one of the problems for the development of effective maternal vaccine against GBS.

### Limitation of the study

The availability of data on GBS serotype distribution in the African countries was limited, with nine studies included to this review. The finding of this study also showed certain heterogeneity although its appearance in the analysis of many studies conducted by different researchers is inevitable.

## Conclusion

The data generated from this systematic review and meta-analysis provided important epidemiological information on colonizing GBS isolated from the 22,206 pregnant women in the 21 African countries. The most antibiotic resistance proportion estimate was observed in the tetracycline followed by penicillin which remains the drug of choice for GBS in the Westerns. *Streptococcus agalactiae* also exhibited considerable resistance to ampicillin and vancomycin which are usually recommended for maternal IAP in the developed countries. Serotype V, III, Ia, Ib, and II were found to be the most prevalent in the Africa that altogether accounted more than 91.8%. Findings of this review will contribute its part in the GBS vaccine development suited for disease prevention and treatment in Africa, as well as the implementation of effective clinical antibiotic usage. The authors recommended that infection and antibiotic resistance control strategies should include GBS as one of the most infective bacteria particularly for newborns delivered from the colonized pregnant women. In contrary to other continents, very few data are available in Africa about the GBS serotype profiles, thus, more data is needed in Africa to support the international community who are working on the GBS vaccine development.
